# Effect of delayed isolation of peripheral blood mononuclear cells on cell viability and functionality

**DOI:** 10.1186/s12865-025-00701-y

**Published:** 2025-03-15

**Authors:** Sarah Lehle, Simon Völkl, Katharina Seitz, Chloë Goossens, Julius Emons, Matthias Ruebner, Sabrina Uhrig, Philipp Ziegler, Anna-Katharin Theuser, Matthias W. Beckmann, Peter A. Fasching, Hanna Huebner

**Affiliations:** 1 Department of Gynecology and Obstetrics, Universitätsklinikum Erlangen, Friedrich-Alexander-Universität Erlangen-Nürnberg (FAU), Erlangen, Germany; 2 Comprehensive Cancer Center Erlangen-EMN (CCC ER-EMN), Erlangen, Germany; 3 Comprehensive Cancer Center Alliance WERA (CCC WERA), Erlangen, Germany; 4 Bavarian Cancer Research Center (BZKF), Erlangen, Germany; 5 Department of Internal Medicine 5, Hematology, and Oncology, Universitätsklinikum Erlangen, Friedrich-Alexander-Universität Erlangen-Nürnberg (FAU), Erlangen, Germany; 6 Institut für Frauengesundheit GmbH (IFG), Erlangen, Germany

**Keywords:** PBMCs, Biobanking, Cell viability, Cytotoxicity, Phase IV clinical trial

## Abstract

**Background:**

Peripheral blood mononuclear cells (PBMCs) are valuable biomarkers, providing crucial insights into the patients’ immune system. Reliable biobanking of PBMCs is essential to minimize heterogeneity. In multicenter trials, blood sample transportation to central laboratories can increase the time between blood collection and PBMC isolation. This study evaluated the effect of prolonged blood hold time on PBMC viability and cytotoxicity.

**Methods:**

From July 2021 to May 2023, 104 patients with early HER2-positive breast cancer were enrolled in the NeoOn trial, of whom 49 patients were included in this subproject. PBMCs were isolated ≤ 6 hours (h) or ≥ 20 h after blood collection. PBMC yield and viability were determined using the LUNA-II Automated Cell Counter. Flow cytometry was used to quantify *in vitro* cytotoxicity, the percentage of natural killer (NK) and T cells, as well as apoptotic and necrotic cells.

**Results:**

Isolating PBMCs ≥ 20 h resulted in a higher cell yield, but lower NK cell viability compared to PBMCs ≤ 6 h. PBMCs ≥ 20 h were less robust to thawing and showed higher loss during recovery. Compared to PMBCS ≤ 6 h, PBMCs ≥ 20 h exhibited lower antibody-mediated cytotoxicity (*p* ≤ 0.0001) and antibody-dependent phagocytosis (*p* < 0.0051). While the percentage of T and NK cells and the T cell viability remained unaffected by hold time, the percentage of apoptotic NK cells was higher for PBMCs ≥ 20 h (41.0 ± 12.9% vs. 23.8 ± 13.4%; *p* = 0.0364).

**Conclusions:**

Extended blood storage time caused increased apoptosis and necrosis of NK cells, adversely affecting PBMC quality and reducing NK cell related functionality. Hence, blood hold time should be minimized to maintain PBMC integrity and NK cell functionality for *in vitro* biomarker assays.

**Trial registration:**

Trial registration number: EudraCT 2020-001943-21. Date of registration: December 29th 2020

**Supplementary Information:**

The online version contains supplementary material available at 10.1186/s12865-025-00701-y.

## Introduction

The analysis of immune cell subsets in cancer therapy, particularly peripheral blood mononuclear cells (PBMCs), has gained increasing significance in recent years due to their potential as biomarkers. In cancer therapy, state-of-the-art treatment strategies include antibodies against tumor surface proteins and immune checkpoint inhibitors that enhance antitumor immune cell functions [1, 2]. PBMCs offer valuable insights into the peripheral immune response patterns, making them easy to obtain and valuable biomarkers for clinical use and precision medicine [3]. Several applications of PBMCs as biomarkers have been described in literature [4–7]. Not only the phenotype of PBMCs but also the activity and functionality of these immune cells are important aspects of current research [8, 9]. These features of PBMCs can be evaluated by cytotoxicity assays [4], cytokine secretion assays [10] and migration assays [11]. In this context, the cytotoxic capacity of PBMCs against tumor cells is a feature, which can be quantified in *in vitro* assays. More specifically, therapeutic antibodies can help immune cells to identify target cells and induce cytolytic activity or phagocytosis. This process is referred to as antibody-dependent cytotoxicity (ADCC) and antibody-dependent phagocytosis (ADCP). Initial results suggest ADCC as a biomarker of response to antibody-based therapy [4, 5].

Working with PBMCs, however, poses logistical challenges, especially in multicenter clinical trials. Blood samples either have to be processed at the study site itself, with quite a huge technical and timely effort, or have to be shipped to a central laboratory unit resulting in a delay of processing [3]. PBMCs are typically isolated using density gradient centrifugation, wherein a Pancoll-Paque density gradient solution is employed to separate mononuclear cells from other blood components [12]. In order to use those cells for addressing immunological research questions, PBMCs are either directly used after isolation or cryopreserved for the use at later time points. Cryopreservation offers a higher logistical flexibility and thus, is often preferred. The biobanking of viable cells is challenging as the sample quality must be maintained to generate reliable data [13]. Ideally, cell phenotype and functionality are preserved and represent the patient’s immune cell characteristics at the time of blood collection. Variations in blood processing protocols across different sites can significantly impact sample quality, affecting the reliability of study results [14]. Factors such as blood collection methods, storage conditions, and processing time are known to influence the PBMC phenotype and functionality, highlighting the need for standardized handling procedures [15, 16]. Most of these factors can be easily controlled, enabling consistent and reliable PBMC quality. In multicenter clinical trials, however, investigators are faced with logistical challenges. To guarantee unified handling and processing of blood samples and to reduce the burden on the individual study sites, blood is often shipped to and processed at a centralized laboratory resulting in a delay between blood draw and PBMC isolation. Due to shipment time, blood usually gets processed with a delay of over 12 hours (h) after blood draw. Phenotypic changes of PBMCs have been described after extended hold time of blood [17]. Flow cytometry analysis revealed reduced levels of chemokine receptors CXCR5, CCR6, and CXCR3 on B cells, reduced levels of T cell immunoreceptor with Ig and ITIM domains (TIGIT) on regulatory memory T cells, and reduced levels of CXCR3 on CD8 positive (CD8+) T cells [17]. Extended blood storage time further results in reduced CD3ζ expression on T cells, a receptor important for T cell function [13] and decreased expression of the chemokine receptors CCR4 and CCR7 on natural killer (NK) cells [18]. In addition to these phenotypic effects, functional changes such as reduced cytokine production (IFN-γ, TNF-α) and decreased degranulation capacity (measured by CD107a expression) have been observed for NK cells [18].

To address these challenges, centralized biobanking facilities play a crucial role in ensuring uniform sample processing and storage. Despite their importance, the impact of prolonged blood hold time before PBMC isolation on cell quality and in particular cell cytotoxicity remains poorly understood. Investigating this aspect is essential for optimizing blood processing protocols and enhancing the quality of bio-samples collected in multicenter trials. Therefore, this study aimed to evaluate the effects of extended blood hold time on PBMC numbers, viability, and functionality, particularly focusing on the cytotoxic capacity when introduced to antibody-based treatments. To this end, PBMCs, collected as part of the single-arm, open-label, multicenter, phase IV NeoOn clinical trial (EudraCT:2020-001943-21) were used. By elucidating these dynamics, we can develop strategies to mitigate logistical challenges and improve the reliability of immune cell-based research in cancer therapy.

## Materials and methods

### Patients

**Fig. 1:**
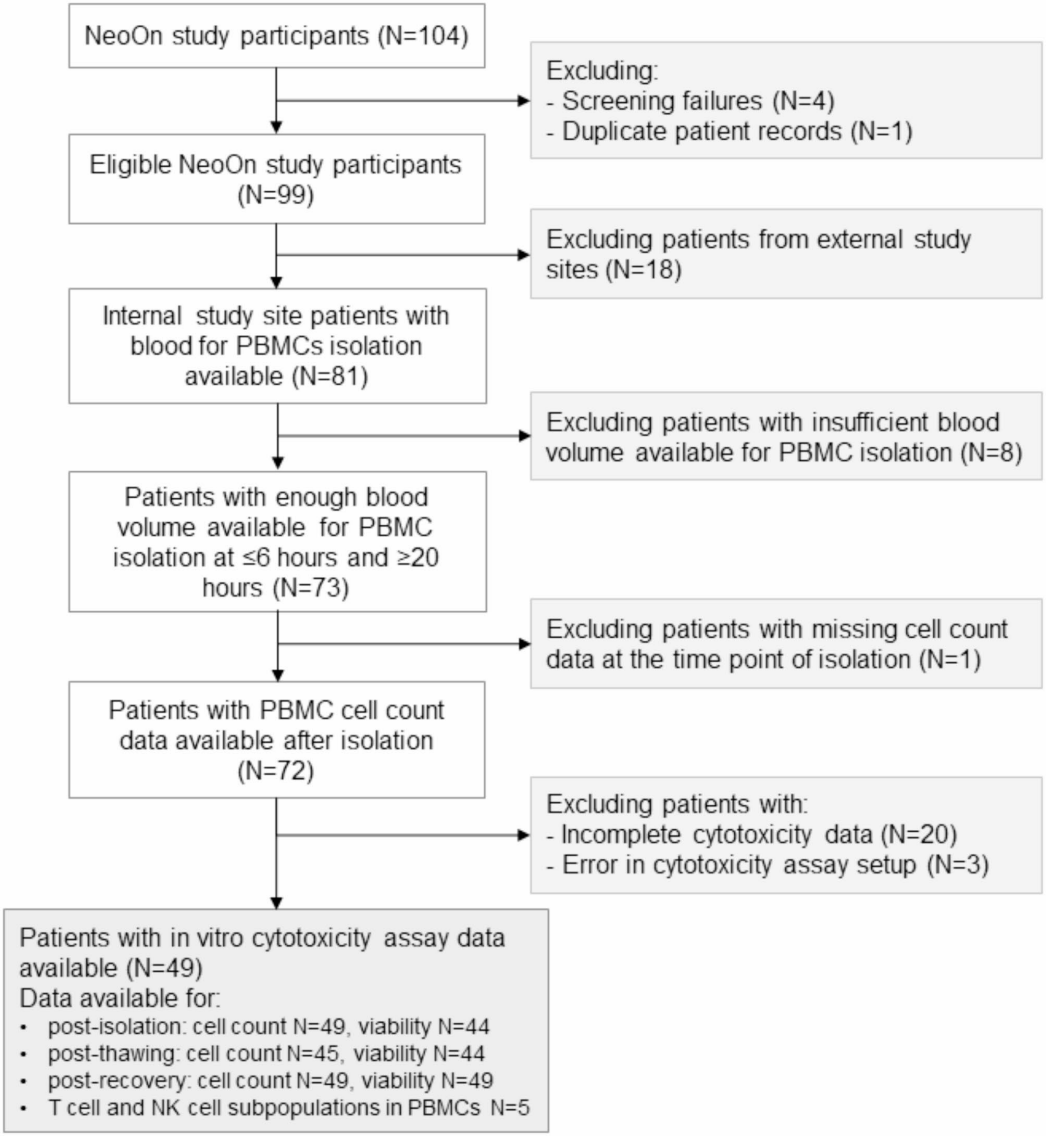
Patient flow chart showing the patient selection criteria for the peripheral blood mononuclear cell (PBMC) analysis in the NeoOn study

Between July 12th 2021 and May 17th 2023, 104 patients with early human epidermal growth factor receptor 2 (HER2)-positive breast cancer were enrolled in the NeoOn study (Fig. 1). The NeoOn study is an open-label, multicenter Phase IV trial evaluating the combination of the trastuzumab biosimilar ontruzant with chemotherapy in the neoadjuvant setting that included patients across 3 study centers in Germany. Trastuzumab and ontruzant are humanized monoclonal antibodies directed against HER2. The NeoOn study and the associated research projects were reviewed and approved by the relevant authority and ethics committee (leading ethical commission: ethical commission of the medical faculty at the Friedrich-Alexander-Universität Erlangen-Nürnberg, Erlangen, Germany; protocol code: 550_20 Az; initial approval: May 14th 2021. Clinical trial number EudraCT:2020-001943-21; registration date December 29th 2020). The study was conducted according to the guidelines of Good Clinical Practice and the Declaration of Helsinki and all patients provided written informed consent. Female adult (≥ 18 years old) patients with histologically confirmed HER2-positive breast cancer, ECOG 0–1, a measurable lesion ≥ 1 cm, indication for chemotherapy, adequate organ function, completed staging without an indication of distant disease and core biopsy available before the first chemotherapy and after the last neoadjuvant study treatment could be included in the NeoOn study. Patients could not be included if they were hypersensitive to ontruzant, received prior chemotherapy, radiation therapy or small molecule therapy for any reason, were pregnant or breastfeeding, had an active infection requiring systemic therapy, had a history of (non-infectious) pneumonitis that required steroids or current pneumonitis, had immunological diseases (active autoimmune disease or other disease that systemic treatment with corticosteroids or immunosuppressive drugs, active or prior primary or acquired immunodeficiency, active or prior inflammatory bowel disease), had a known history or positive antibody test for any of the following infections: human immunodeficiency virus (HIV), history of acute or chronic hepatitis B or hepatitis C, had known congestive heart failure > NYHA I and/or coronary heart disease, angina pectoris, previous history of myocardial infarction, uncontrolled or poorly controlled arterial hypertension (e.g. blood pressure > 160/90 mmHg under treatment with two or more antihypertensive drugs), rhythm disorders with clinically significant valvular heart disease, had Pre-existing motor or sensory neuropathy of a severity grade ≥ 2 by National Cancer Institute Common Terminology Criteria for Adverse Events (CTCAE) v4.03.

HER2 and hormone receptor status were defined according to ASCO/CAP guidelines [19, 20]. Estrogen receptor and progesterone receptor status were defined as positive if ≥ 1% was stained, while a positive HER2 status required an immunohistochemistry score of 3 + or positive fluorescence in situ hybridization/chromogenic in situ hybridization. Blood was collected in BD Vacutainer EDTA tubes (BD Biosciences, New Jersey, USA) at baseline prior to therapy. The total of 104 patients were included into the NeoOn trial (Fig. 1). For the subproject assessing PBMC quality, screen failures (*N* = 4) and duplicate patient entries (*N* = 1) were excluded. Furthermore, patients recruited from an external study site were excluded (*N* = 18) due to shipment delay of PBMCs. Of the 81 patients recruited at the central study site, only those with sufficient blood volume available for PBMC isolation on both (A) the day of blood draw and (B) 20 h after blood draw, were included in the analysis (*N* = 73). Furthermore, the analysis was limited to patients for whom both PBMC yield after isolation and cytotoxicity assay data were available, resulting in a final sample size of *N* = 49.

### Isolation and cryopreservation of peripheral blood mononuclear cells (PBMCs)

PBMCs were isolated at two timepoints after blood draw. PBMCs were isolated within 1 to 6 h after blood collection from one tube (referred to as ≤ 6 h PBMCs) or between 20 h and 30 h after blood collection from two EDTA tubes (referred to ≥ 20 h PBMCs). Blood for the ≥ 20 h PBMC isolation was stored stationary until isolation. The specific timeframes resulted from the clinical and biobanking routine. Blood samples from patients were received mainly before 1 p.m (8 a.m. to 1 p.m.), and PBMC isolation was conducted at fixed timepoints. The primary isolation batch was processed at 2 p.m., while blood samples arriving after 2 p.m. were processed in a secondary batch at 6 p.m. For PBMC isolation, blood was diluted 1:1 with Dulbecco´s Phosphate Buffered Saline (Sigma-Aldrich, St. Louis, USA). Next, a maximum volume of 35 ml diluted blood was gently overlayed onto 15 ml of a Pancoll-Paque (PAN Biotech, Aidenbach, Germany) and centrifuged at 900 x g for 17 minutes (min) at room temperature with the brake off. After centrifugation, distinct layers of plasma, PBMCs, Pancoll-Paque and red blood cells formed. Using a 10 ml serological pipet, the PBMCs were collected by gentle aspiration and placed in a fresh 50 ml tube. Cells were washed twice by filling the tube with up to 45 ml Hanks´ Balanced Salt Solution (Gibco, Waltham, USA) medium and centrifuging at 400 x g for 10 min at room temperature. The PBMC cell pellet was resuspended in RPMI 1640 (Sigma Aldrich, St. Louis, USA), counted with a LUNA-II Automated Cell Counter (Logos Biosystems, Dongan-gu Anyang-si, South Korea), and stored in 1 ml freezing medium consisting of RPMI 1640 (Sigma Aldrich, St. Louis, USA), supplemented with 40% fetal bovine serum (Sigma Aldrich, St. Louis, USA) and 20% dimethyl sulfoxid (Sigma Aldrich, St. Louis, USA) for cryopreservation at a cell number 5 × 10^6^ cells per aliquot. The cell counter has a standard deviation of 2.44 × 10^3^ cells/ml and a coefficient of variance of 0.5% quantifying replicate dilutions. The cryovials were frozen in a CoolCell LX Freezing Container (Corning, New York, USA) at an intermediate cooling stage of -80 °C before storage in liquid nitrogen for up to one month. The median storage duration for PBMCs until further use in the respective cytotoxicity assays was 20.00 days with a range of 3.00 to 29.00 days in the ≤ 6 h group and 18.00 days with a range of 2.00 to 28.00 days in the ≥ 20 h group. According to the literature, ADCC from frozen PBMCs can be reliably assessed from frozen PBMCs up to one month of cryopreservation [21].

### PBMC thawing and recovery

The thawing and recovery of matching ≤ 6 h and ≥ 20 h PBMC samples were performed in parallel by the same scientist to ensure consistency. The cryovials were thawed for 3 min in a 37 °C water bath. Cryovials from each patient were combined and washed with an equal volume of PBS to achieve a cell number of > 1 × 10^7^. After centrifugation for 10 min at 400 x g, the PBMCs were quantified using a LUNA-II Automated Cell Counter (Logos Biosystems, Dongan-gu Anyang-si, South Korea). To recover, the thawed PBMCs were cultured overnight for 12 to 14 h in FACS tubes (Fisher Scientific, Hampton, USA). During recovery PBMCs were maintained in RPMI 1640 (Sigma Aldrich, St. Louis, USA) supplemented with 10% fetal bovine serum (Sigma Aldrich, St. Louis, USA), 1% Penicillin Streptomycin (Life Technologies, Carlsbad, USA), 1 mM Sodium pyruvate (Life Technologies, Carlsbad, USA), 2 mM L-Glutamine (Life Technologies, Carlsbad, USA) and non-essential amino acids (Life Technologies, Carlsbad, USA). Using the automated cell count data the change in cell number after thawing was calculated as: cell number post thawing/ cell number post isolation. Similarly, the cell change during overnight recovery was calculated as: cell number post recovery/ cell number post thawing. A value of one indicates no change in cell number while a value below 1 indicates a decrease in cell number during thawing/ overnight recovery.

### Cell line

The human HER2-positive breast cancer cell line SKBR3 (catalogue number ATCC HTB-30, ATCC, Manassas, VA, USA) was cultured at 37 °C and 5% CO_2_ and maintained in RPMI 1640 media supplemented with 10% fetal bovine serum (Sigma Aldrich, St. Louis, USA) and 1% Penicillin Streptomycin (Life Technologies, Carlsbad, USA). Cells were tested negative for *Mycoplasma* contamination.

### Antibody dependent cell-mediated cytotoxicity (ADCC) and antibody dependent cell-mediated phagocytosis (ADCP) assay

**Fig. 2:**
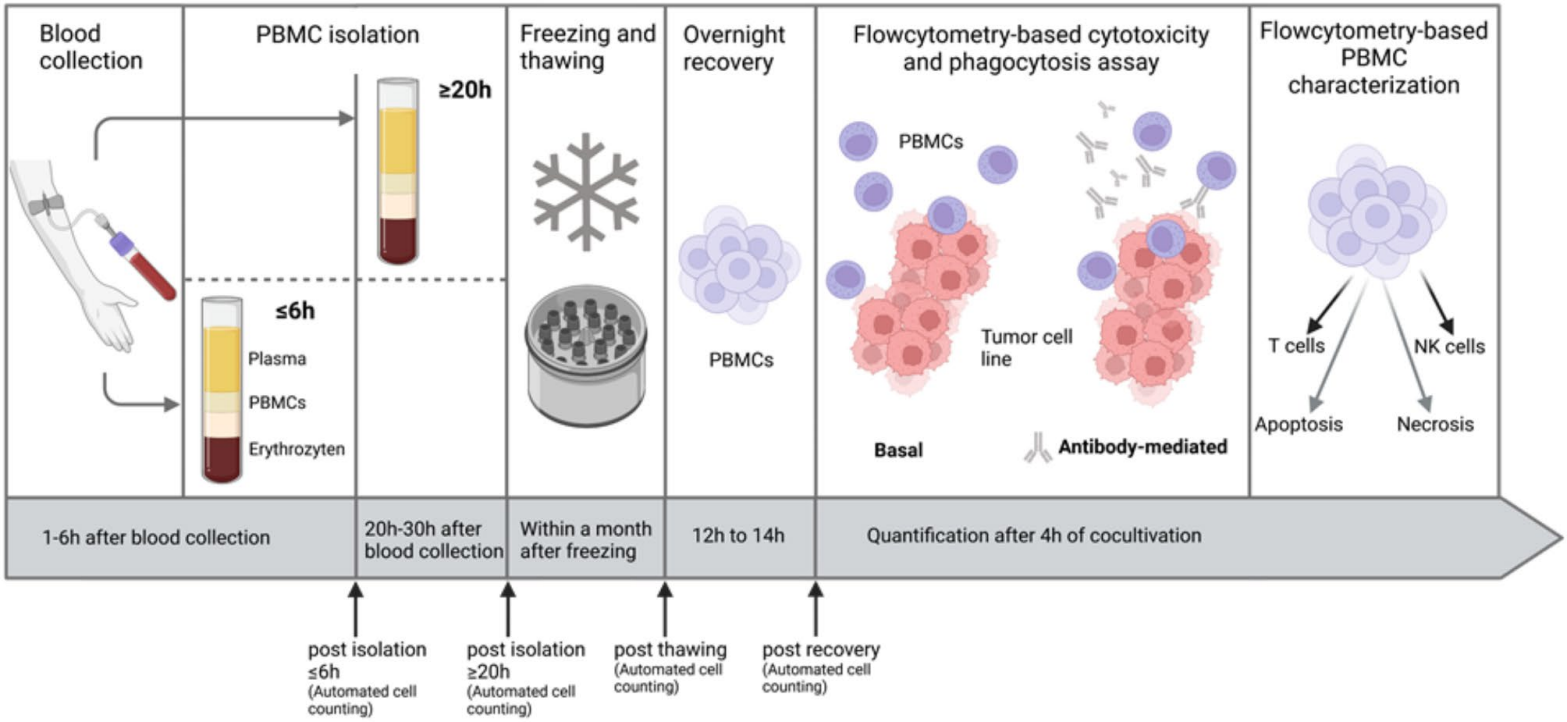
Timeline for peripheral blood mononuclear cells (PBMC) isolation and analysis. Created with BioRender.com

The ADCC and ADCP assays were all run by the same scientist and processed in parallel for matching ≤ 6 h and ≥ 20 h PBMC samples to ensure consistency. After overnight recovery, concentration and viability were determined using the LUNA-II Automated Cell Counter. PBMCs were co-cultured with the HER2-positive cell line SKBR3 at an effector to target ratio of 50:1 (1 × 10^6^ effector cells and 2 × 10^4^ target cells) for 4 h. The term effector cells refers to the PBMCs, which are composed of T cells, B cells, NK cells, monocytes and dendritic cells, all of which exhibit immunological effector functions. The tumor cell line used in the *in vitro* assay to trigger an immune reaction is referred to as the target cells. Cytotoxicity without treatment and after addition of the antibody ontruzant (10 µg/ml) were determined in separate tubes. Antibody concentration was adopted from established ADCC assays based on the literature [5, 8]. To quantify the different cell populations by flow cytometry, SK-BR3 cells were stained with carboxyfluorescein succinimidyl ester (CFSE), (Sigma Aldrich, St. Louis, USA) and PBMCs were stained with anti-CD45 APV-H7 antibody (clone 2D1; Biolegend, San Diego, USA). Target cells were stained at a concentration of 4 µM for 10 min. Dead cells were identified by 7-Aminoactinomycin D (7AAD)-staining (BD Biosciences, New Jersey, USA). In brief, the gating strategy was the following: the time parameter was used to ensure instrument stability. Doublets were excluded by forward scatter-area / forward scatter height. Next, the CD45- and CFSE+ cell population was defined as target cells. Cytotoxicity was evaluated as percentage of dead target cells (7AAD+). The CD45+ CFSE- gate, which was defined as leukocyte gate, was used to determine vital leucocytes presented as 7AAD-. In the vital leucocyte gate the CFSE+ CD45+ cell population was defined as phagocytosed cells [22]. Gating was performed by the same scientist in one batch to promote homogeneous gating. A detailed presentation of the gating strategy can be found in the additional file 1. Samples with less than 30% of CD45+ cells (CD45+ CFSE-) after co-cultivation were excluded from analysis (Figs. 1 and 2). A small cohort of patient PBMCs (*N* = 5) were further analyzed for necrosis and early apoptosis and the percentage of T and NK cells was quantified by flow cytometry. Flow cytometric analyses were performed as described previously [23]. In short, cells were stained with 7AAD (BD Biosciences, New Jersey, USA) and annexin V FITC(BD Biosciences, New Jersey, USA), anti-human anti-CD3 BV510 (clone UCHT1; BD Biosciences, New Jersey, USA), anti-CD56 BV421 (clone MY31; BD Biosciences, New Jersey, USA), according to the manufacturer’s protocol. The gating strategy was defined as follows: Doublets were excluded by FSC-A/FSC-H, cells were gated for CD45, monocytes were excluded by FSC-A/SSC-A, T cells were gated by CD3+ and CD56-, and NK cells were gated by CD3- and CD56+. T cells and NK cells were subdivided in apoptotic (Annexin V+/7AAD-), necrotic (Annexin V+/7AAD+) and viable (Annexin V-(7AAD-)) cells. Flow cytometry data were acquired on a 5-laser LSR Fortessa (BD Biosciences, New Jersey, USA) and data analysis was performed using Kaluza software v2.1 (Beckmann Coulter, Krefeld, Germany).

### Statistical analysis

For plotting graphs and statistical analysis, Graph Pad Prism version 9.0.2 (GraphPad software, San Diego, CA, USA) was used. Parametric model assumptions were evaluated by a quantile–quantile plot and a Shapiro-Wilk test. According to the p-values calculated by the Shapiro-Wilk test the distribution of the data was deemed parametric or non-parametric. Mean values between two groups were compared using a paired Student´s t-test for normally distributed data and a Wilcoxon test for non-parametric analysis. Paired analyses were performed exclusively on data where measurements were available for both the ≤ 6 h and the ≥ 20 h samples from the same patient. If data were missing for one time point in a pair, the patient was excluded from the paired analysis. Relationships between two variables were assessed using Pearson´s correlation coefficient (r). All tests were two-sided and *p* values ≤ 0.05 were considered statistically significant.

## Results

The study included 49 female breast cancer patients, of whom patient characteristics are depicted in Table 1. Patients had with a mean age of 51.5 ± 12.3 years and a BMI of 25.3 ± 5.0 (Table 1). 26 patients were pre/perimenopausal and 23 patients were postmenopausal. 34 patients had hormone receptor-positive disease, while 15 had hormone receptor-negative disease (Table 1).

**Table 1:** Patient and tumor characteristics

Characteristic	
Patients n (%)	49 (100.0)
Age, mean ± SD, years	51.5 ± 12.3
BMI, mean ± SD	25.3 ± 5.0
Menopausal status	
Pre/perimenopausal, n (%)	26 (53.1)
Postmenopausal, n (%)	23 (46.9)
Unknown, n (%)	0 (0.0)
HR	
Negative, n (%)	15 (30.6)
Positive, n (%)	34 (69.4)
Unknown, n (%)	0 (0.0)
cT	
cT1, n (%)	17 (34.7)
cT2-cT4, n (%)	31 (63.3)
Unknown, n (%)	1 (2.0)
Grading	
G1-G2, n (%)	18 (36.7)
G3, n (%)	30 (61.2)
Unknown, n (%)	1 (2.0)

SD = Standard deviation; BMI = body mass index; HR = hormone receptor status; cT = clinical staging

**Fig. 3:**
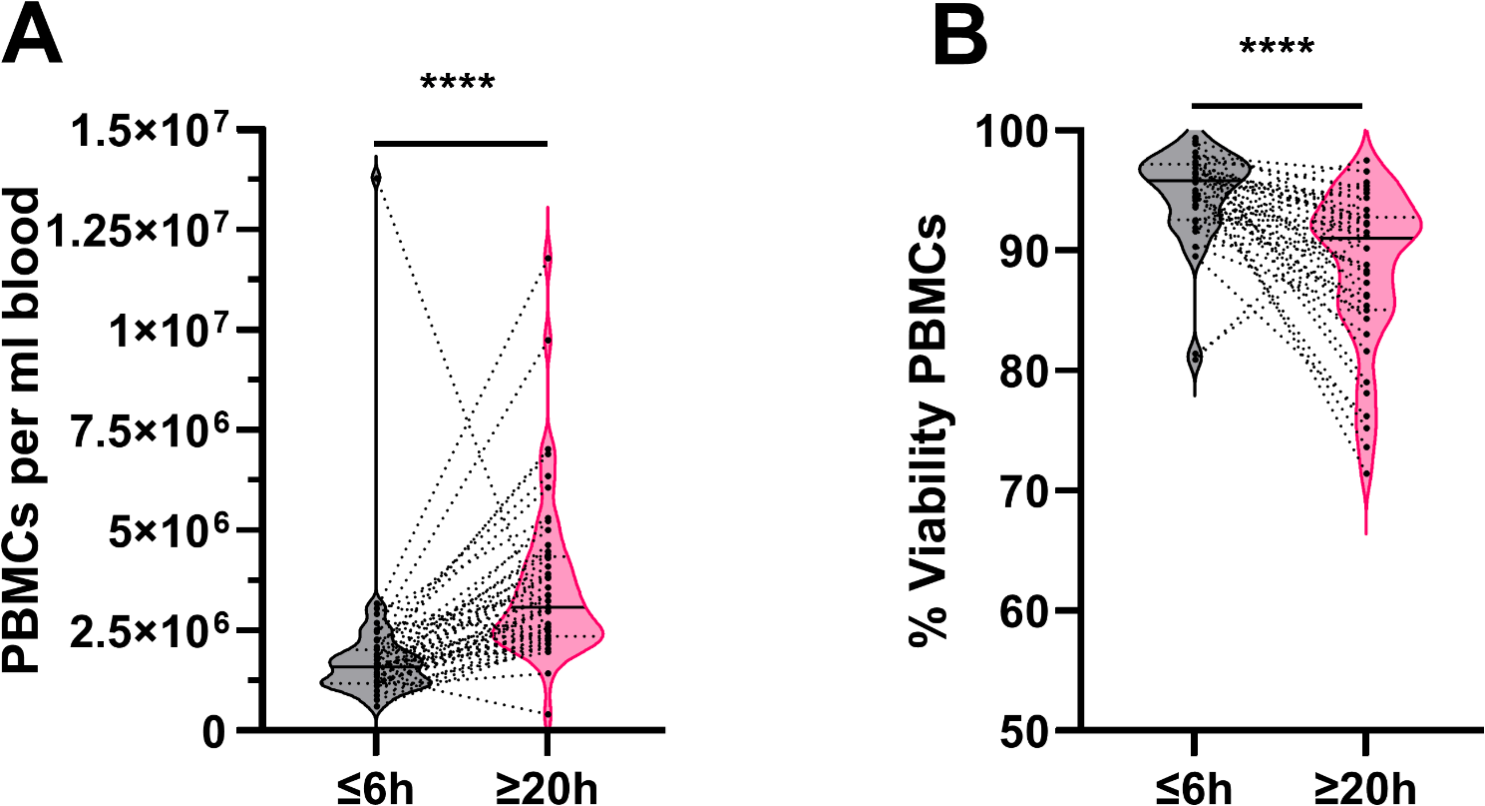
The yield and viability of peripheral blood mononuclear cells (PBMCs) isolated within 6 h of blood collection (≤ 6 h) and 20 h (≥ 20 h) after collection were compared. Viability was assessed using trypan blue dye. (**A**) Number of PBMCs per ml blood and (**B**) % viability after isolation. Cell counts and viability were determined by the Luna II Automated Cell Counter. Paired analyses were conducted only for samples with data available at both time points. Patients with missing data at one time point were excluded from the respective paired analysis. The central line in the violin plot represents the median value and the dashed line are the 25 and 75 percentiles. (**A**) *N* = 49, (**B**) *N* = 44. Data distribution was non-parametric. P-value was calculated using a Wilcoxon Test. ****, *p* ≤ 0.0001

The PBMC yield after Pancoll-Paque isolation was evaluated for each patient using blood samples collected within 6 h on the day of blood draw (timepoint ≤ 6 h) and 20 h later (timepoint ≥ 20 h). The number of PBMCs per ml of blood was higher when collected after 20 h compared to within 6 h (*p* < 0.0001; ≥ 20 h 3.1 × 10^6^ ± 2.0 × 10^6^ cells/ml blood; ≤ 6 h 1.6 × 10^6^ ± 1.8 × 10^6^ cells/ml blood) (Fig. 3A). In terms of viability, PBMCs isolated after 20 h exhibited a lower percentage of viable cells (*p* < 0.0001; ≥ 20 h 91.0 ± 6.5%; ≤ 6 h 95.8 ± 3.9%) (Fig. 3B).

**Fig. 4:**
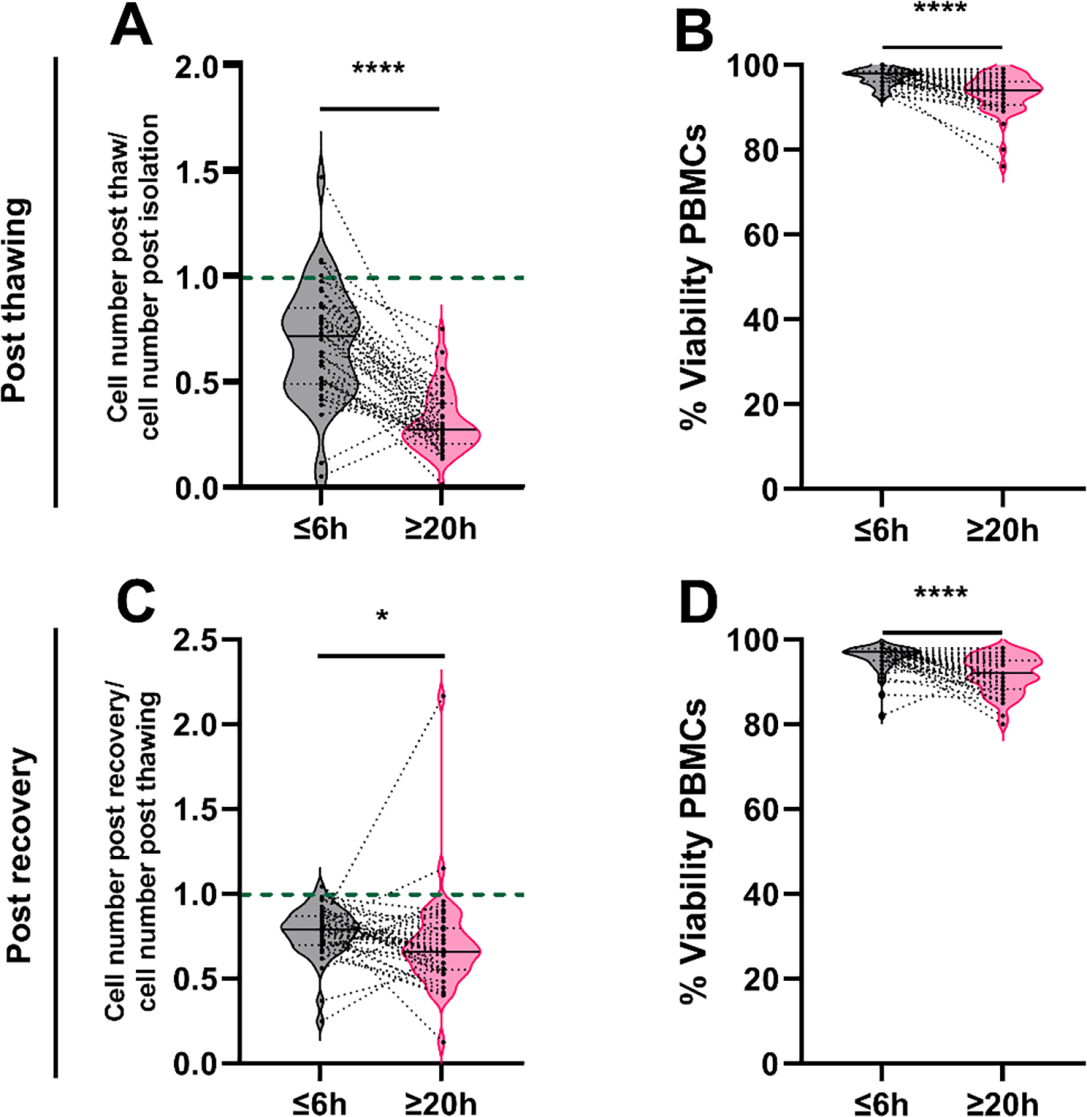
Change of cell number and viability post thawing (**A**, **B**) and post recovery (**C**, **D**) of peripheral blood mononuclear cells (PBMC) isolated within 6 h after blood draw (≤ 6 h) and 20 h after blood collection (≥ 20 h). (**A**) Ratio of PBMC cell number after thawing (post thaw) to PBMC cell number after isolation (post isolation) and (**B**) % viability after thawing. (**C**) Ratio of PBMC cell number after overnight recovery (post recovery) to PBMC cell number after thawing (post thawing) and (**D**) % viability after recovery. Cell counts and viability were determined using the Luna II Automated Cell Counter. Paired analyses were conducted only for samples with data available at both time points. Patients with missing data at one time point were excluded from the respective paired analysis. The dashed green line marks a ratio of 1, representing an unchanged cell number. The central line in the violin plot represents the median value and the dashed line are the 25 and 75 percentiles. (**A**, **B**) *N* = 45, (**C**, **D**) *N* = 44. Data distribution was non-parametric. P-value was calculated using a Wilcoxon Test. *, *p* ≤ 0.05, ****, *p* ≤ 0.0001

PBMCs isolated after 20 h showed a lower post thaw to post isolation cell number ratio (*p* < 0.0001; ≥ 20 h 0.3 ± 0.1; ≤ 6 h 0.7 ± 0.3; Fig. 4A). PBMCs isolated within 6 h had a higher viability after thawing compared to those isolated after 20 h (*p* < 0.0001; 98.0 ± 2.0% viability ≤ 6 h; 94.0 ± 4.6% viability ≥ 20 h) (Fig. 4B). Similar but less pronounced trends were observed following overnight recovery. PBMCs isolated after 20 h showed a lower post recovery to post thawing ratio (*p* < 0.0001; ≥ 20 h 0.7 ± 0.3; ≤ 6 h 0.8 ± 0.1; Fig. 4C) as well as a lower viability (*p* < 0.0001; 97.0 ± 3.2% ≤ 6 h; 92.0 ± 4.4% ≥ 20 h) (Fig. 4D).

**Fig. 5:**
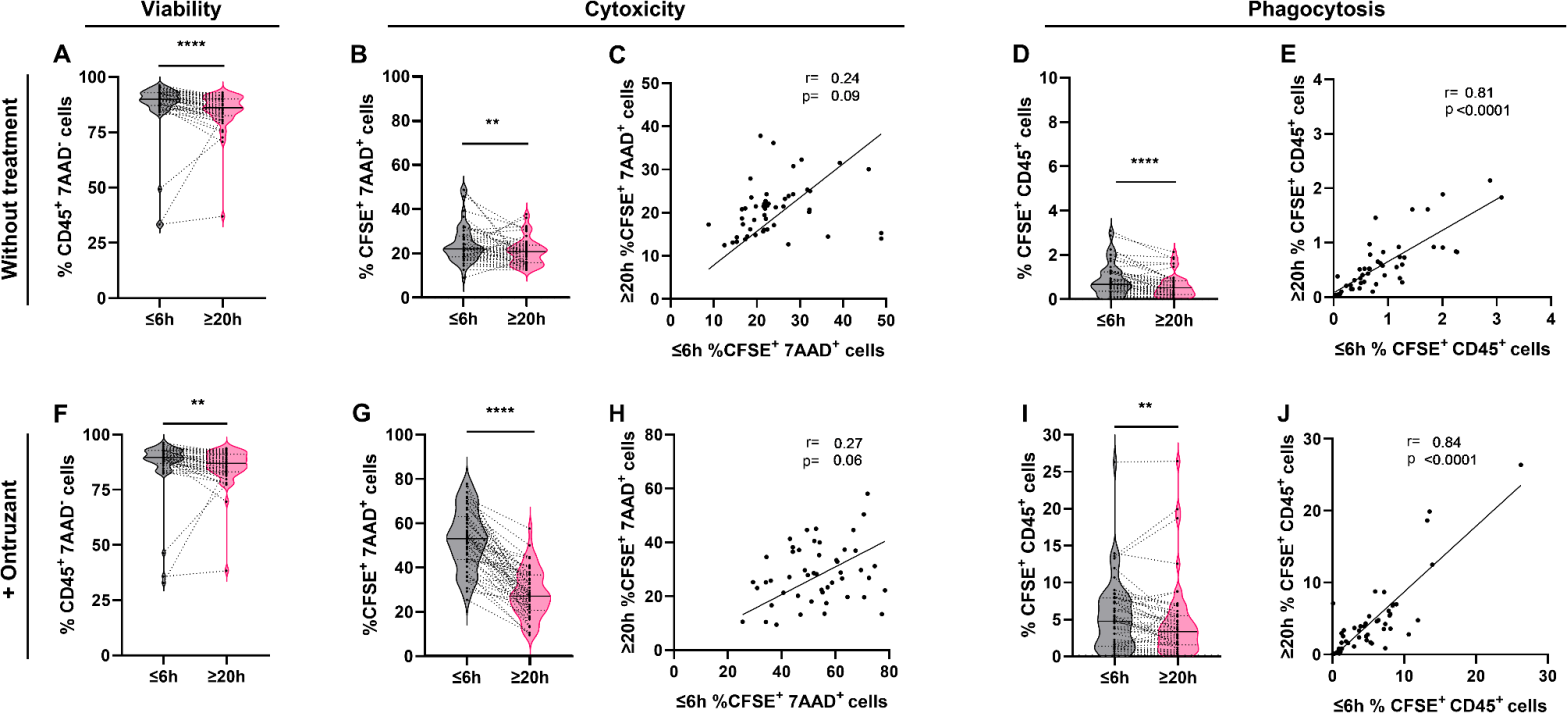
Viability, cytotoxicity, and phagocytosis without treatment, as well as ontruzant-mediated cytotoxicity and phagocytosis were evaluated in peripheral blood mononuclear cells (PBMCs) isolated within 6 h after blood collection (≤ 6 h) and 20 h after blood collection (≥ 20 h). (**A**, **F**) The percentage of vital PBMCs was defined as the cell population in the (CD45+ 7-Aminoactinomycin D-) gate. (**B**, **C**, **D**, **E**) The cytotoxicity and phagocytosis of PBMCs was assessed without and (**G**, **H**, **I**, **J**) after adding ontruzant, visualized as violin plots. The central line in the violin plot represents the median value and the dashed line are the 25 and 75 percentiles. Correlations of cytotoxicity and phagocytosis for 6 h PBMCs and 20 h PBMCs were evaluated without (**C**, **E**) and with antibody (**H**, **J**) and visualized in scatter plots. Paired analyses were conducted only for samples with data available at both time points. Patients with missing data at one time point were excluded from the respective paired analysis. The solid black line in the correlation represents the linear regression. Statistical comparison was performed using a (**A**, **B**, **D**, **F**, **I**) Wilcoxon test for non-parametric data and a (**G**) t-test for parametric data. *N* = 49. **, *p* ≤ 0.01; ****, *p* ≤ 0.0001

In the flow cytometry-based cytotoxicity assay without treatment, a smaller cell population of PBMCs remained viable (CD45+ 7AAD-) when isolated after 20 h compared to isolation within 6 h (*p* < 0.0001; ≤6 h 89.9 ± 13.0% viability; ≥ 20 h 86.2 ± 8.7% viability) (Fig. 5A). Cytotoxicity and phagocytosis assessed after co-cultivating PBMCs with the HER2 + cell line SKBR3 for 4 h without treatment, were significantly lower in PBMCs isolated after 20 h compared to isolation within 6 h (*p* < 0.0051; *p* < 0.0001) (Fig. 5B, D). The mean cytotoxicity of the ≤ 6 h samples was 22.0 ± 8.6% and 20.8 ± 6.1% of the ≥ 20 h PBMCs samples (Fig. 5B). The mean phagocytosis for the ≤ 6 h samples was 0.7 ± 0.7% and 0.5 ± 0.5% for the ≥ 20 h samples (Fig. 5D). Significant correlation for phagocytosis but not cytotoxicity was observed between PBMCs isolated before 6 h and after 20 h (*p* < 0.0001) (Fig. 5C, E). Furthermore, the ability of the antibody ontruzant to enhance the killing of target cells was evaluated. The viability of ≥ 20 h PBMCs in the ontruzant mediated cytotoxicity/phagocytosis assay was lower compared to the viability of ≤ 6 h PBMCs (*p* = 0.0016; ≤ 6 h 89.6 ± 13.0% viability; ≥ 20 h 86.9 ± 8.6% viability) (Fig. 5F). The use of PBMCs isolated 20 h after blood draw resulted in a significant lower ontruzant mediated ADCC compared to PBMCs isolated within 6 h (Fig. 5G). Specifically, the percentage of 7AAD+ target cells decreased from 53.0 ± 13.5% for ≤ 6 h PBMCs to 27.1 ± 10.9% for ≥ 20 h PBMCs (*p* < 0.0001). Similar trends were demonstrated for ontruzant mediated phagocytosis, with a reduction in 7AAD+ target cells from 4.8 ± 4.8% for ≤ 6 h PBMCs to 3.4 ± 5.2% for ≥ 20 h PBMCs (*p* < 0.0029) (Fig. 5I). The cytotoxicity of PBMCs after addition of ontruzant did not correlate between PBMCs isolated within 6 h and after 20 h, while the phagocytosis of PBMCs after addition of ontruzant correlated between PBMCs isolated within 6 h and after 20 h (*p* < 0.0001) (Fig. 5H, J).

**Fig. 6:**
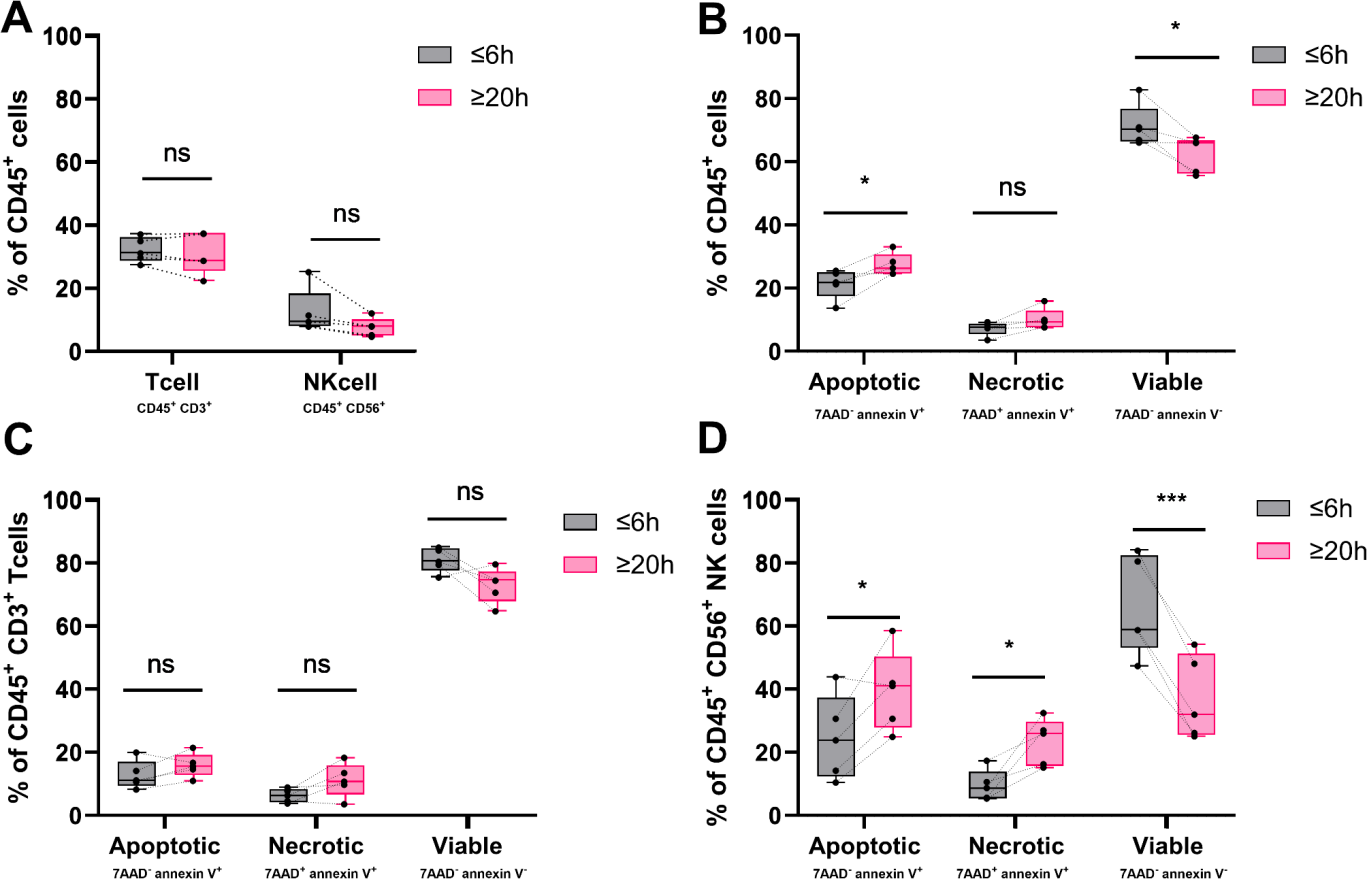
The viability and percentage of T and natural killer (NK) cell subsets in peripheral blood mononuclear cells (PBMCs) with different blood hold times. PBMCs were defined as all CD45 + cells. (**A**) The percentage of T cells (CD3+) and NK cells (CD56+) was determined in PBMCs isolated within 6 h (≤ 6 h) and after 20 h (≥ 20 h) after blood collection using flow cytometrie. The viability (7-Aminoactinomycin D- (7AAD)annexin V-), apoptotic (7AAD- annexin V+) and necrotic (7AAD + annexin V+) subset of PBMCs (**B**), T cells (CD3+) (**C**) and NK cells (CD56+) (**D**) were determined. Data is presented as box plots with median and interquartile range with whiskers representing the Tukey method. Paired analyses were conducted only for samples with data available at both time points. Patients with missing data at one time point were excluded from the respective paired analysis. P-value for T cell distribution (**A**) was calculated using a t test while NK-cell comparision was calculated with a wilcoxon test due to non-parametric distribution of the data. (**B**, **C**, **D**) p-values were calculated by a t-test. *N* = 5; *, *p* ≤ 0.05; ***, *p* ≤ 0.001

The viability of NK and T cell subsets in the PBMCs of a subgroup of patients (*N* = 5) was characterized using flow cytometric analyses (Fig. 6). PBMCs isolated 20 h after blood collection did not differ in the composition of NK and T cell populations compared to PBMCs isolated within 6 h of collection (Fig. 6A). However, there was an increase in the percentage of apoptotic cells from 21.8 ± 4.7% of ≤ 6 h PBMCs to 26.3 ± 3.5% of ≥ 20 h PBMCs (*p* = 0.0311) (Fig. 6B). The percentage of viable PBMCs also decreased from 70.3 ± 6.7% for ≤ 6 h PBMCs to 66.0 ± 5.7% for ≥ 20 h PBMCs (*p* = 0.0364). There was no difference in the subset of necrotic PBMCs between ≤ 6 h and ≥ 20 h PBMCs. T cell viability, the percentage of apoptotic and necrotic cells did not differ between ≤ 6 h and ≥ 20 h PBMCs (Fig. 6C). The subset of NK cells in PBMCs isolated 20 h after blood collection exhibited higher rates of apoptosis (*p* = 0.0410; ≤ 6 h 23.8 ± 13.4%; ≥ 20 h 41.0 ± 12.9%) and necrosis (*p* = 0.0189; ≤ 6 h 8.7 ± 4.9%; ≥ 20 h 26.0 ± 7.4%) (Fig. 6D). Consistently, the percentage of viable NK cells in PBMCs isolated within 6 h was higher compared to PBMCs isolated after 20 h (*p* = 0.0004; ≤ 6 h 58.8 ± 15.7%; ≥ 20 h 32.0 ± 18.5%).

## Discussion

Our study demonstrates that the timing of PBMC isolation impacts mainly cell yield, viability of NK cells, and general functionality. We found that isolating PBMCs within 6 h of blood collection results in superior quality, including higher viability of some subsets as well as reduced cell loss during thawing and overnight recovery. In contrast, PBMCs isolated after 20 h exhibited lower NK viability and general functionality, particularly in cytotoxicity and phagocytosis assays.

Increased PBMC yield after prolonged storage of blood was reported in previous studies. For example, one study compared PBMC isolation yields after holding blood for 3 h and 18 h in seven patients and found a trend towards increased cell counts [24]. The analyzed patient cohorts, however, were small and significant effects could not be detected [24, 25]. Nonetheless, an increase in cell number could be explained by granulocyte contamination as described previously [13, 16, 17, 26]. Extended hold time of blood activates granulocytes, resulting in decreased density and subsequent enrichment of granulocytes in the PBMC fraction [16]. Granulocyte contamination has a negative impact on sample quality, as granulocytes are short-lived cells, that release DNA upon death, resulting in clumps in the sample [26]. The formation of aggregates may result in cell loss and reduced cell function [26], which is why some research groups suggest DNAse treatment of the samples [26]. An alternative to prevent granulocyte contamination in the first place, is diluting blood samples with PBS prior to prolonged storage [13]. Although very cost efficient, this solution requires sterile blood sample handling at the external study centers which cannot be easily implemented. In multicenter trials where prolonged blood hold times cannot be avoided it is recommended to implement quality control protocols for PBMCs that take granulocyte contamination into account.

Further, our results show, that PBMCs isolated within 6 h after blood collection are of superior quality for several reasons. PBMCs isolated within 6 h of blood draw had less cell loss during thawing and recovery and a slightly better viability during isolation, post thawing and after recovery. However, the absolute viability difference of viability between 6 h and 20 h PBMCs was consistently less than 5%. Given that according to the manufacturer the automated cell counter has a reproducibility of ± 2.09 × 10^4^ cells/ml and a coefficient of variance of 3.62%, the 5% change in viability may not be relevant in practice. More striking was the change in cell number during thawing calculated as cell count post thawing/cell count post isolation from 0.7 for 6 h PBMCs to 0.3 for 20 h PBMCs, indicating a loss of 40% of cells. These parameters are important to know before designing assays to ensure that the yield of viable cells meets the requirements of a functional assay. There are mixed observations regarding the viability as described in the literature. Jerram et al. described a significant reduction of the total percentage of viable cells for PBMCs from around 80% viability for instantly isolated PBMCs to approximately 55% for PBMCs isolated 6 h and 45% for PBMCs isolated 24 h after venipuncture [17]. Yi et al. also reported decreasing PBMC quality with delayed blood processing, as evidenced by a deterioration in the quality of Pancoll-Paque density gradient separation and red blood cell contamination [16]. Additionally, a reduced number of lymphocytes with increase in processing delay was observed in this study [16]. Furthermore, a small study described a trend of decreasing PBMC viability, post thaw recovery as well as post thaw viability from approximately 98% (6 h) to 95% (32 h) with increasing blood processing delay [25]. However, there are some groups which were not able to detect a difference in PBMC viability after a delay in blood processing. Posevitz-Fejfar et al., who compared PBMC yield and viability from PBMC isolated from 2 h vs. 24 h holded blood specimens in nine healthy patients. And the group of Navas et al. described a slight but non-significant reduction in the frequency of viable cells from a viability over 90% to a viability over 85% after 24 h in eight healthy patients [27]. The missing effect in these studies could however be due to the small sample size that increased the likelihood of a type II error.

Viability of cells is an important aspect if functional assays are planned which include such PBMCs. The capacity of PBMCs to mediate antibody-based cytotoxicity and phagocytosis is an important aspect within current research questions. Here we showed that the functional capacity was reduced in PBMCs with prolonged storage before isolation. PBMCs isolated after prolonged storage of blood were less active towards antibody-based stimuli. Interestingly, the capacity to induce phagocytosis of ≤ 6 h PBMCs and ≥ 20 h PBMCs showed a significant correlation, suggesting that despite the overall decrease in capacity after prolonged blood hold time, the functionality might still be relevant for biomarker assays as the patient specificity is maintained. Although the effect size was not very high the mean value of phagocyted cells was significantly lower in ≥ 20 h PBMC samples compared to the matched ≤ 6 h samples.In contrast, the cytotoxicity of PBMCs from these two groups did not show a significant correlation, indicating that the impact on cytotoxic effects might be more pronounced and relevant. Our findings align with previous studies that have investigated the impact of delayed blood processing on PBMC functionality. For instance, one study demonstrated that delayed blood processing negatively affects NK cell function, as evidenced by degranulation and cytokine secretion [18]. One group evaluated the performance of NK cells in a cytotoxicity assay on PBMCs isolated the same day and the day after blood collection in a cohort of 27 patients [28]. For the functional evaluation a chromium release cytotoxicity assay on thawed PBMCs that were co-cultivated with K562 target cells for 4 h was performed [28]. In this setting the cytotoxicity of PBMCs isolated on day two correlated with day one but showed a trend to decreased cytotoxicity [28]. The investigators analyzing PBMC cytotoxicity in ADCC and ADCP assays usually report blood hold times between 0 h and 8 h after venipuncture [29–31], thus experience about blood hold effects in these types of assays is limited. Additionally, another study reported reduced expression of CD3ζ, a marker for T cell function, following prolonged blood storage [13]. Minimizing the granulocyte contamination in PBMCs by cell sorting or by diluting the blood with PBS prior to storage increased CD3ζ expression and improved T cell proliferation [13]. This suggests, that extended storage of blood is not the central factor causing impaired PBMCs function, but the granulocyte contamination which can be avoided by blood dilution.

Previous studies have shown that blood storage leads to neutrophil activation, though does not affect the PBMC subset distribution [17]. In our study, we demonstrated that prolonged blood storage did not alter the composition PBMCs in regards to NK cell and T cell numbers. However, there are also findings from the literature describing a reduction of the NK cell fraction in PBMC samples exceeding 24 h of blood hold time [18]. The limited number of patients in the published literature indicates the need for further investigation [17, 18]. We observed that NK cells were more sensitive to extended hold time than T cells reflecting in an increased apoptosis and necrosis rate. The reduced number of viable NK cells could also partly explain the reduction of cytotoxic capacity when blood is stored for a longer time period.

Our study has several limitations that should be noted. Firstly, our conclusions are limited to EDTA blood. It is possible that blood collection tubes containing other stabilizing agents may have a different effect on blood and PBMC viability, which was not evaluated in our study. Secondly, the sample size for T and NK cell subset analysis was relatively small, increasing the risk of type II error, where real differences may not have been detected. Thirdly, while we observed significant differences in PBMC yield and functionality between 6 h and 20 h, the clinical relevance of these findings needs further exploration. Another limitation of our study was the ADCP assay. The assumption that only phagocytosis was quantified should be treated with caution, since the assay did not include specific staining for macrophages/monocytes, which are the main mediators of phagocytosis. Nevertheless, the strategy to define the phagocytosis gate as cells with a double positive staining (positive for tumor and immune cell staining) was pursued by others in the literature before [32, 33]. Still, precautions were taken in the gating to exclude tumor cells that were superficially bound to the immune cells in the phagocytosis gate. A proportion of the doublets were sorted out in the FSC-H and the FSC-A gates (Additional Fig. 1B). Based on the assumption that after being phagocytosed the CFSE signal would decrease, we excluded CD45+ cells with high CFSE signal, which might be tumor cells bound to immune cells (Additional Fig. 1D). On the other hand, CD45+ cells with low CFSE signal were excluded by leaving a gap between CD45+ and CFSE- and the CD45+ and CFSE+ gate (Additional Fig. 1G). This strategy was chosen to ensure that immune cells with a low CFSE signal due to other reasons were excluded. Overall, the steps should ensure the validity of the phagocytosis gating strategy. Additionally, our study focused on a specific patient population suffering from early breast cancer, and the results may not be generalizable to other patients or healthy individuals.

## Conclusions

In conclusion, our findings highlight the importance of timely blood sample processing in ensuring high quality of PBMCs for subsequent applications. The holding time of EDTA blood impacts cell viability of NK cells and the cytotoxic capacity of PBMCs. To ensure consistent sample quality in clinical trials, it is important to track the time between blood collection and PBMC isolation. Clear documentation allows for the establishment of cut-offs and the maintenance of homogenous sample quality. When planning a multi-center clinical trial, it might be unavoidable to use blood that has been shipped before processing resulting in a delayed PBMC isolation. In these cases, reduced cytotoxic activity in functional assays caused by the higher fraction of apoptotic and necrotic NK cells has to be expected. The results underscore the need for standardized protocols in clinical and research settings to address these issues. Future research should focus on understanding the mechanisms behind these differences and exploring potential methods to preserve PBMC integrity during extended storage. By overcoming these challenges, we can enhance the reliability and consistency of PBMC-based assays, thereby improving their application in both research and clinical practice.

## Electronic supplementary material

Below is the link to the electronic supplementary material.

Supplementary Material 1

## Data Availability

Data used for all analyses that support the findings of this study are available from the corresponding author, H.H., upon reasonable request.

## Abbreviations

7AAD: 7 Aminoactinomycin D
ADCC: antibody dependent cytotoxicity
ADCP: antibody dependent phagocytosis
BMI: body mass index
CD8+: CD8-positive
CFSE: carboxyfluorescein succinimidyl ester
cT: clinical staging
H: hours
HER2: human epidermal growth factor receptor 2
HR: hormone receptor
Min: minutes
NK: natural killer
PBMC: peripheral blood mononuclear cells
SD: standard deviation
TIGIT: T cell immunoreceptor with Ig and ITIM domains
