# Supplementary material for: Effect of delayed isolation of peripheral blood mononuclear cells on cell viability and functionality
Source: BMC Immunol. 2025 Mar 15;26:21. doi: 10.1186/s12865-025-00701-y (PMC11909936; doi:10.1186/s12865-025-00701-y)
Supplement: Supplementary file 1 — Supplementary Material 1 [file 12865_2025_701_MOESM1_ESM.docx]

Additional file


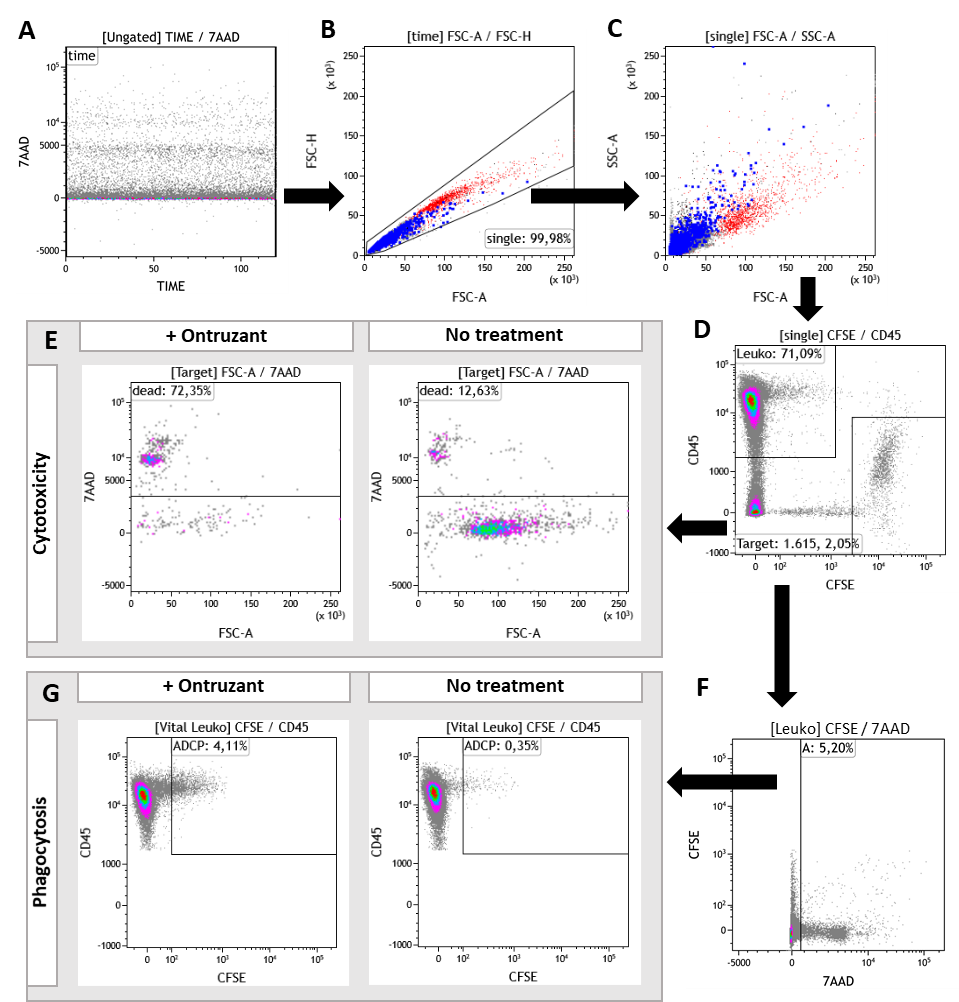


**Additional figure 1.** Gating strategy for ADCC and ADCP Assay. (**A**) The time parameter allowed to monitor instrument stability. (**B**) Cell singlets were selected via forwards scatter height (FSC-H) and forward scatter area (FSC-A).(**C**) Cells were visualized in the sideward scatter area (SSC-A) and the FSC-A. (**D**) Target cells were determined by CD45- and CFSE+ gate and leukocytes by the CD45+ and CFSE- gate. The CD45+ CFSE+ gate with strong CFSE signal was excluded from further analysis. Cells with strong CD45+ and CFSE+ signal are likely to be leukocytes bound to tumor cells. For phagocytosis we assumed the CFSE signal decreased. (**E**) Cytotoxicity was defined as dead target cells determined as 7AAD+ cells in the target cell gate. Cytotoxicity was determined in two separate FACS tubes one containing ontruzant (10µg/ml) and one without treatment. (**F**) Vital leukocytes were determined as 7AAD- in the leucocyte gate. (**G**) Phagocytosis was defined as CFSE+ and CD45+ cells in the vital PBMC fraction. The vital PBMCs gate was chosen since dead PBMCs are assumed to be irrelevant for phagocytosis. A gap between CD45+ and CFSE- and the CD45+ and CFSE+ gate was left to exclude CD45+ cells with weak CFSE signal due to other reasons. Phagocytosis was evaluated in two separate FACS tubes one with ontruzant and one without treatment.
